# Protein *S*-palmitoylation regulates the virulence of plant pathogenic fungi

**DOI:** 10.1128/mbio.03472-24

**Published:** 2024-12-17

**Authors:** Mengmeng Guo, Leeza Tariq, Fengming Song

**Affiliations:** 1 Ministry of Agriculture Key Laboratory of Molecular Biology of Crop Pathogens and Insect Pests, Institute of Biotechnology, Zhejiang University, Hangzhou, China; 2 Key Laboratory of Biology of Crop Pathogens and Insects of Zhejiang Province, Institute of Biotechnology, Zhejiang University, Hangzhou, China; 3 National Key Laboratory for Rice Biology and Breeding, Institute of Biotechnology, Zhejiang University, Hangzhou, China; Duke University Hospital, Durham, North Carolina, USA

**Keywords:** palmitoyltransferase, *S*-palmitoylation, *Ustilaginoidea virens*, virulence

## Abstract

Protein *S*-palmitoylation, a universal posttranslational modification catalyzed by a specific group of palmitoyltransferases, plays crucial roles in diverse biological processes across organisms by modulating protein functions. However, its roles in the virulence of plant pathogenic fungi remain underexplored. In a recent study, Y. Duan, P. Li, D. Zhang, L. Wang, et al. (mBio 15:e02704-24, 2024, https://doi.org/10.1128/mbio.02704-24) reported that the palmitoyltransferases UvPfa3 and UvPfa4 regulate the virulence of the rice false smut pathogen *Ustilaginoidea virens*. Through comprehensive characterization of *S*-palmitoylation sites, they revealed that *S*-palmitoylated proteins in *U. virens* are enriched in mitogen-activated protein (MAP) kinase and autophagy pathways, with MAP kinase UvSlt2 being a key target of UvPfa4-mediated *S*-palmitoylation. Further investigation demonstrated that *S*-palmitoylation of UvSlt2 is critical for its kinase activity, substrate interaction ability, and virulence function in *U. virens*. These findings reveal UvPfa4-mediated *S*-palmitoylation as a vital regulatory mechanism in *U. virens* virulence, highlighting the importance of protein *S*-palmitoylation in the pathogenicity of plant pathogenic fungi.

## COMMENTARY

Posttranslational modifications significantly expand the functional diversity and complexity of proteins, with many playing pivotal roles in regulating growth, development, stress responses, and pathogenicity in plant pathogenic fungi (1). *S*-Palmitoylation, also called *S*-acylation, is a notable example of such modifications. This reversible form of protein lipidation is catalyzed by a group of palmitoyltransferases (PATs) and removed by depalmitoylases including acyl protein thioesterases (2, 3). It influences protein functions by modulating protein stability, trafficking, enzymatic activity, protein-protein interactions, and protein-membrane associations (2, 4, 5).

Direct and indirect evidence suggests that *S*-palmitoylation is involved in various biological processes of fungi, including virulence. For example, deletion of the PAT gene *Pfa4* impaired growth and reduced the virulence of *Cryptococcus neoformans* (6, 7). Similarly, treatment with *S*-palmitoylation inhibitor 2-bromopalmitate (2-BP) or deletion of the PAT gene *AnAkrA* affects hyphal morphogenesis, conidiation, stress response, and virulence in *Aspergillus fumigatus* and *Aspergillus nidulans* (8–10). In watermelon Fusarium wilt pathogen *Fusarium oxysporum* f. sp. *niveum* (*Fon*), four out of six *FonPAT* genes are required for virulence, with FonPAT2 *S*-palmitoylating core subunits of adaptor protein complex 2 (FonAP-2)—FonAP-2α, FonAP-2β, and FonAP-2μ—to ensure its proper assembly and function in virulence (11). Despite these findings, the mechanistic role of *S*-palmitoylation in the virulence of plant pathogenic fungi remains poorly understood. Recently, Duan et al. (12) reported that the PAT genes *UvPfa3* and *UvPfa4* are essential for the virulence of the pathogenic ascomycetous fungus *Ustilaginoidea virens* and demonstrated that UvPfa4-mediated *S*-palmitoylation is critical for the function of mitogen-activated protein (MAP) kinase UvSlt2 in fungal virulence.

*U. virens* causes rice false smut, a highly devastating grain disease that leads to severe yield losses and the production of noxious mycotoxins (13). The authors first demonstrated that 2-BP significantly inhibited *S*-palmitoylation in the mycelia of *U. virens*, leading to the suppression of growth, development, and virulence (12). These findings suggest that *S*-palmitoylation plays a critical role in various biological processes in *U. virens*, including its virulence. A subsequent proteomic screen utilizing an affinity-directed mass spectrometry approach identified 4,089 *S*-palmitoylation sites from 2,192 proteins, including several virulence-related proteins involved in autophagy and MAP kinase signaling pathways. Notably, *S*-palmitoylation commonly occurs at specific cysteine residues (14). The identified *S*-palmitoylated proteins in *U. virens* displayed a shared common sequence motif, in which lysine, glutamic acid, and aspartic acid are frequently present within 10 amino acids flanking the central *S*-palmitoylation cysteine.

PATs are typically encoded by a small gene family; for instance, yeast encodes seven PATs (15). The *U. virens* genome encodes six PATs (12), consistent with the number found in *Fon* (11). Functional analyses of deletion mutants revealed that the *U. virens* PAT genes have distinct roles in vegetative growth, asexual reproduction, and responses to abiotic stress. Particularly, the Δ*UvPfa3* mutant exhibited a complete loss of virulence, while the Δ*UvPfa4* mutant showed a significant reduction in virulence on rice. Both mutants also showed substantial reductions in overall *S*-palmitoylation levels, with 120 and 88 downregulated *S*-palmitoylated proteins, respectively. These results establish a direct connection between *S*-palmitoylation and virulence in *U. virens*. Interestingly, UvPfa3 in *U. virens* and FonFAT1 in *Fon* are phylogenetically related to yeast ScPfa3 and both are crucial for virulence (11, 12), implying that these PATs may be evolutionarily conserved in plant pathogenic fungi for virulence.

MAP kinase signaling pathways contribute to fungal virulence by phosphorylating various substrate proteins during infection-related development (16). Comparative proteomic analyses of *S*-palmitoylated proteins in Δ*UvPfa3* and Δ*UvPfa4* mutants, compared to the wild-type strain, revealed that several components of MAP kinase signaling pathways were *S*-palmitoylated. Particularly, UvPfa4 was found to interact with *S*-palmitoylate UvSlt2, confirming that UvSlt2 is a substrate of UvPfa4. UvSlt2 harbors two *S*-palmitoylation sites at C161 and C166, and mutations at these sites completely abolished its *S*-palmitoylation in palmitoylation-dead variant UvSlt2^C161A/C166A^. Similar to the Δ*UvPfa4* mutant, Δ*UvSlt2* mutant lost its virulence on rice, integrating UvPfa4 and UvSlt2 into a virulence regulatory module. However, UvSlt2^C161A/C166A^ did not completely lose its virulence function; it partially restored the virulence defect of the Δ*UvSlt2* mutant on rice, suggesting that *S*-palmitoylation is important but not entirely required for the full function of UvSlt2 in *U. virens* virulence. This is further supported by the similar ability of UvSlt2^C161A/C166A^ in complementation of the defects in vegetative growth and asexual reproduction observed in the Δ*UvSlt2* mutant.

*S*-Palmitoylation affects protein stability, activity, membrane localization, and protein-protein interactions, thereby modulating protein functions (2, 4, 5). The *S*-palmitoylation-dead variant UvSlt2^C161A/C166A^ exhibited decreased kinase activity and interaction ability with its substrate UvRlm1, although its subcellular localization remained unchanged. The Δ*UvRlm1* mutant also lost virulence on rice, suggesting that the UvPfa4-UvSlt2-UvRlm1 module positively regulates *U. virens* virulence. Further investigation revealed that *S*-palmitoylation at C161 and C166 alters the structure of UvSlt2. While the UvSlt2^C161A/C166A^ variant had a nearly identical structure to the unmodified UvSlt2, with no changes in the hydrophobic solvent-accessible surface area, the *S*-palmitoylated UvSlt2^C161palm/C166palm^ displayed a markedly different structure with increased hydrophobic solvent-accessible surface area that likely enhances the binding capacity to its substrates. However, despite the unchanged hydrophobic surface area, UvSlt2^C161A/C166A^ showed reduced interaction with UvRlm1. It remains unknown whether the UvSlt2^C161A/C166A^ variant reduces its ability to phosphorylate UvRlm1.

Extensive research has focused on posttranslational modifications such as phosphorylation and ubiquitination; however, less attention has been paid to the functions of different types of protein lipidation, including *S*-palmitoylation, in regulating the virulence of plant pathogenic fungi. Duan et al. (12) provided key biochemical and proteomic evidence supporting *S*-palmitoylation as a crucial mechanism in fungal virulence, introducing a novel regulatory pathway involving UvSlt2-mediated MAP kinase signaling in *U. virens* virulence. However, several open questions need further investigation. First, substrate specificity varies among PATs (17, 18); for instance, *Fon* FonPAT1 and FonPAT2 exhibit different *S*-palmitoylation activities on substrates such as the core subunits of FonAP-2 complex (11). Similarly, *U. virens* PATs, UvPfa4 and UvPfa3, may regulate distinct pathways through selectively *S*-palmitoylating specific substrates. Second, while proteomic analyses have identified a large number of *S*-palmitoylated proteins (11, 12), further research is needed to elucidate the *S*-palmitoylation patterns of virulence-related substrates, offering a broader understanding of its role in fungal pathogenicity. Third, since *S*-palmitoylation is a reversible posttranslational modification (2, 3), understanding how it is dynamically regulated under different environmental conditions and during host interactions could reveal new insights into its function in pathogenicity. Finally, *S*-palmitoylation might represent an adaptative strategy by fungi to cope with environmental changes and host defense mechanisms, highlighting its potential as a target for antifungal agents. Therefore, the development of S-palmitoylation inhibitors could pave the way for innovative strategies in crop disease management.
